# Non-Myopic Beam Scheduling for Multiple Smart-Target Tracking in Phased Array Radar Networks

**DOI:** 10.3390/s24237755

**Published:** 2024-12-04

**Authors:** Yuhang Hao, Zengfu Wang, José Niño-Mora, Jing Fu, Quan Pan, Min Yang

**Affiliations:** 1 School of Automation, Northwestern Polytechnical University, Xi’an 710072, China; yuhanghao@mail.nwpu.edu.cn (Y.H.); quanpan@nwpu.edu.cn (Q.P.); sdta_ym717@mail.nwpu.edu.cn (M.Y.); 2 Key Laboratory of Information Fusion Technology, Ministry of Education, Xi’an 710072, China; 3 Departamento de Estadística, Universidad Carlos III de Madrid, Calle Madrid, 126, 28903 Getafe, Madrid, Spain; jose.nino@uc3m.es; 4 School of Engineering, RMIT University, Melbourne, VIC 3000, Australia; jing.fu@rmit.edu.au

**Keywords:** target tracking, beam scheduling, restless bandits, Whittle index

## Abstract

This paper addresses beam scheduling for tracking multiple smart targets in phased array radar networks, aiming to mitigate the performance degradation in previous myopic scheduling methods and enhance the tracking performance, which is measured by a discounted cost objective related to the tracking error covariance (TEC) of the targets. The scheduling problem is formulated as a restless multi-armed bandit problem, where each bandit process is associated with a target and its TEC states evolve with different transition rules for different actions, i.e., either the target is tracked or not. However, non-linear measurement functions necessitate the inclusion of dynamic state information for updating future multi-step TEC states. To compute the marginal productivity (MP) index, the unscented sampling method is employed to predict dynamic and TEC states. Consequently, an unscented sampling-based MP (US-MP) index policy is proposed for selecting targets to track at each time step, which can be applicable to large networks with a realistic number of targets. Numerical evidence presents that the bandit model with the scalar Kalman filter satisfies sufficient conditions for indexability based upon partial conservation laws and extensive simulations validate the effectiveness of the proposed US-MP policy in practical scenarios with TEC states.

## 1. Introduction

State-of-the-art radar scheduling approaches have enabled higher flexibility for tracking smart targets through phased array radar networks, where real-time beam direction control is handled electronically [1,2,3,4]. Dynamic tracking of smart targets, which have the ability to be aware that they are being tracked and adapt their dynamics accordingly to hinder tracking accuracy, has drawn recent attention [5,6,7]. It is recognized that beam scheduling in radar networks with a trade-off between frequency of tracking and probability of maneuvering is complicated and of importance in smart-target tracking. Hence, myopic radar scheduling approaches, where the objective function considers only the current or next step [8], may lead to imprudent tracking decisions. In contrast, non-myopic approaches take into account the accumulative function values of subsequent steps [9,10]. This paper addresses a new model to minimize the expected total discounted error for tracking smart targets through the non-myopic dynamic beam scheduling.

For smart targets, non-myopic radar scheduling problems become substantially more complex, as such targets react to radar sensing by switching their dynamics between different modes, e.g., a constant velocity (CV) mode, a constant acceleration (CA) mode, a constant turn (CT) mode, etc. [5,6,7]. In such scenarios, it might be beneficial to refrain from sensing too often those targets that are likely to hide or escape when tracked, as this would make them harder to track in the future. In [5], the tracking problem was formulated as a Markov decision process (MDP) with an infinite time horizon, which was addressed through a two-stage reinforcement-learning approach with separated optimizations for detection and tracking. In [6], a modified algebraic Riccati equation (MARE) was applied in a Kalman-based target tracking system with smart targets. This work considered game-theoretic methods that aimed to optimize waveform parameters and radar modes under imperfect measurement information. In [7], a partially observable Markov decision process (POMDP) model [11] with multiple dynamic models was considered to minimize the interception risk. A roll-out method based on unscented sampling (US) was introduced to approximate the long-term reward and to select sensors based on the closest distance policy. In [12], based on a POMDP model, the posterior Cramér–Rao lower bound (PCRLB) was used as system state and multi-step cost prediction over the US. An improved decision tree search algorithm leveraging branch and bound was used to achieve non-myopic scheduling optimization for maneuvering target tracking. The POMDP-based branch-and-bound algorithm was applied to sensor allocation problems for enhanced long-term performance in [13,14,15]. Unfortunately, for multi-target tracking, a POMDP model is established with joint states and combinatorial actions. In general, the action space exhibits worst-case exponential complexity. As a result, computing optimal scheduling policies becomes intractable.

A body of work has instead aimed to design suboptimal heuristic index policies with low computational complexity. The optimal resource management problems and index policies are addressed through the restless multi-armed bandit problem (RMABP) model [16], which is an extension to the multi-armed bandit problem (MABP) [17,18,19]. The RMABP focuses on selecting a maximum of *K* from a pool of N≥K *projects* (or bandits), which are versatile entities capable of being either active (i.e., chosen) or passive at each time. While optimally solving RMABP is generally intractable [20], Whittle proposed in [16] a heuristic index policy, which has since been widely applied [21]. The Whittle index is implicitly defined as the state-dependent critical subsidy for passivity that makes both actions optimal in a single-project subproblem. Niño-Mora computed the Whittle index through an explicitly defined marginal productivity (MP) index [22]. Yet, Whittle conjectured that, when all projects are indexable, this index policy should approach optimality asymptotically under the average criterion as *N* and *K* grow to infinity in a fixed ratio [23]. Niño-Mora further developed PCL-indexability conditions, named after satisfaction of partial conservation laws (PCLs) [22,24,25,26]. The work of [22] guarantees the optimality of threshold policies as well as the indexability of the model and its Whittle index was given by an explicitly defined MP index.

The RMABP formulation of multi-target tracking was first considered in [27], extending work in [28]. The work of [27,28] addressed the greedy index policy and defined a greedy index for each target, a scalar function of its tracking error variance (TEV) state, and giving higher tracking priority at each time to targets with larger index values. The work of [29] addressed the multi-target tracking with the scalar Kalman filter and defined the MP index in TEV states. See also [30] for an application for multi-target tracking with jamming and non-detections. A heuristic approach was developed in [31], where scalar-state project approximations were considered by taking the trace of the channel estimation mean square error (MSE) as the system state. In [32], the problem of hunting hiding targets was formulated as an RMABP, where the state of a target is its posterior probability of being exposed. However, in the more practically relevant case of RMABP models with a multi-dimensional state Kalman filter, indexability remains an open problem. Additionally, the Whittle index policy has not yet been studied in practical multi-target tracking scenarios.

Substantial research challenges persist in RMABP-based smart-target tracking. (1) In models with a multi-dimensional continuous tracking error covariance (TEC) state, the application of the Whittle index policy is at present elusive. (2) Tracking actions will increase the probability of targets employing evasive maneuvers. Meanwhile, in practice, the detection probability of the radar is not one, which complicates the assessment of long-term decision-making performance. (3) The non-linear measurement function complicates the update of TEC states, which requires dynamic state information. When computing the MP-based Whittle index, the currently unknown dynamic states for future steps make it impossible to calculate long-term marginal benefits for TEC states.

This paper considers a beam scheduling problem for tracking multiple smart targets to minimize the multi-target tracking error measured by the sum of the traces of TECs over an infinite time horizon. In the vein of the interacting multiple models (IMM) method, the TEC state and dynamic state of each target are updated based on dynamic model probability vectors. The Whittle index policy based on RMABP formulation is exploited to treat each target as a restless project. For multi-dimensional TECs, the MP index policy uses the trace of the TEC state as a threshold and computes the long-term marginal benefits of different actions through a threshold policy. The US method is employed to sample measurements, and the TEC states and dynamic states over future steps under different actions are updated through the non-linear filter. Consequently, an unscented sampling-based MP (US-MP) index policy is proposed for the multi-dimensional TEC state case. Yet, such a goal is hindered by the fact that no proof of indexability is at present available for this model. This paper partially circumvents this difficulty by presenting some numerical evidence supporting the conjecture that the RMAB model with scalar TEV states satisfies the aforementioned PCL-indexability conditions, and uses the proposed US-MP index policy resulting from them. Through extensive simulation results, the proposed index policy is shown to outperform myopic baseline policies in the discounted TEC cost and root mean square error (RMSE) metrics.

The contributions of this paper are summarized as follows.

- The beam scheduling problem is formulated as an infinite-horizon discounted RMABP. The smart targets are characterized by tracking-action-dependent dynamic models with high maneuvering, which will hide themselves when being tracked. Each target is associated with a restless project, whose TEC states and dynamic states evolve through a non-linear filter based on the dynamic model probability vector.
- In the practical case with multi-dimensional TECs, the trace of the TEC state is used as a threshold. Due to the non-linear measurement function, the TEC states under actions can be only updated based on target measurements. We predict TEC states and dynamic states based on the US method when calculating MP indices. Then, the US-MP index policy is proposed.
- For the scalar TEV states, in cases with realistic parameters, we numerically verify PCL-indexability, which leads to Whittle indexability, of the target-tracking problem. The effectiveness of the proposed US-MP index policy for the multi-dimensional TEC state case is also assessed by a simulation study, where it is shown to outperform myopic baseline policies.

The remainder of this paper is organized as follows. In Section 2, the target dynamic models and measurement model are defined. Then, the TEC state update under different actions is introduced and the scheduling problem is formulated. In Section 3, the application of the Whittle index policy is discussed for scalar state cases. The beam selection scheme is developed in the RMABP model based on US-MP indices for practical tracking cases. The computational method of the lower bound of optimization functions and the computational complexity of policies are presented. In Section 4, scalar and multi-dimensional state cases are considered, and reckless and cautious targets with different dynamic model probabilities are also considered. The simulation results demonstrate the superiority of the proposed MP index policies. Section 5 concludes the paper.

## 2. Model Description and Problem Formulation

The target TEC states are estimated using a non-linear filter along with multiple dynamic models to capture the motion characteristics of smart targets. Then, the beam scheduling problem is formulated as an RMABP, of which each bandit process is associated with a target.

### 2.1. Target Dynamic and Measurement Models

**Figure 1:**
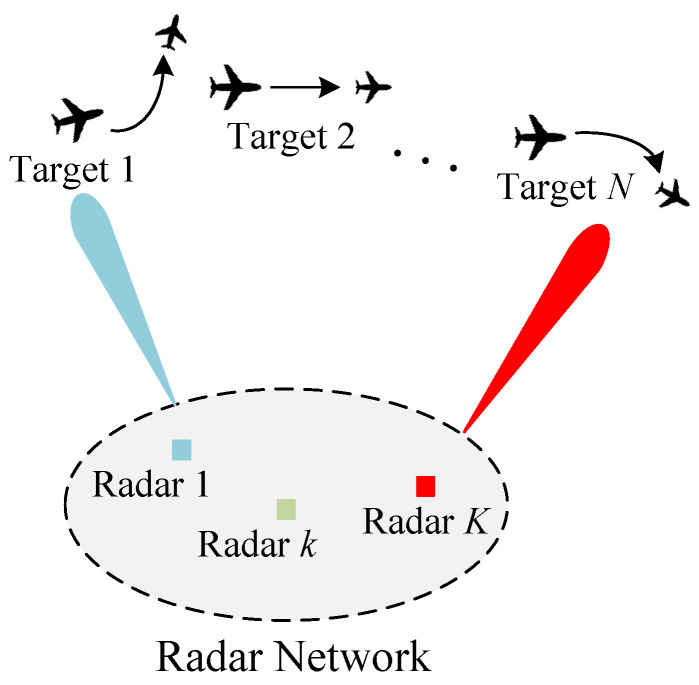
System model of a phased array radar network.

Consider *N* smart targets labeled by n=1,2,…,N and a radar network consisting of K<N phased array radars, as illustrated in Figure 1. Target 1 and Target *N* will likely change their dynamic models in response to radar tracking, while Target 2 will be likely to maintain its dynamic model. All radars are synchronized to operate over time slots t=0,1,…. The radar network centrally steers beams to track at most *K* targets at each time, as one radar beam can be only assigned to one target in a time slot.

Without loss of generality, let the dynamic state of target *n* at time *t* be $x_{n,t}≜{\left[x_{n,t},{\dot{x}}_{n,t},y_{n,t},{\dot{y}}_{n,t}\right]}^{\prime }\in R^{4}$, where ${\left[x_{n,t},y_{n,t}\right]}^{\prime }$ is the position, and ${\left[{\dot{x}}_{n,t},{\dot{y}}_{n,t}\right]}^{\prime }$ is the velocity. ${\left[\cdot \right]}^{\prime }$ is the transpose operator. Targets follow independent and linear dynamics. Meanwhile, they are smart, that is, they react to radar radiation by switching between *M* dynamic models, labeled by m=1,2,…,M, e.g., CV, CA, CT, etc. Target *n*’s dynamics under model *m* is

(1) $$x_{n,t}=F_{n}^{m}x_{n,t-1}+w_{n,t-1}^{m},$$

where $F_{n}^{m}$ is the state transition matrix, and $w_{n,t-1}^{m}$ is process noise with mean being zero and covariance matrix being $Q_{n}^{m}$. Define $Q_{n}^{m}=q_{n}^{m}Q_{n}$ for a covariance matrix $Q_{n}$, where $0<q_{n}^{1}<q_{n}^{2}<\cdots <q_{n}^{M}$, and m=1 corresponds to the CV model, so the uncertainty of the dynamic model increases with *m*.

At each time *t*, a binary variable $a_{n,t}\in \left\{0,1\right\}$ denotes the tracking action imposed on target *n*. Let $a_{n,t}=0$ if target *n* is not tracked; $a_{n,t}=1$ otherwise. Let the detection probability of the radars be $p_{d}\in \left[0,1\right]$ [33]. If target *n* is detected at time *t*, a measurement $z_{n,t}$ is derived with the following measurement equation,

(2) $$z_{n,t}=H_{n}\left(x_{n,t}\right)+v_{n,t},$$

where $H_{n}\left(x_{n,t}\right)=\left(r_{n,t}\left(x_{n,t}\right),\theta_{n,t}\left(x_{n,t}\right)\right)$ with the range $r_{n,t}\left(x_{n,t}\right)$ and the azimuth $\theta_{n,t}\left(x_{n,t}\right)$ being defined as

(3) $$\begin{array}{c} r_{n,t}\left(x_{n,t}\right)=\sqrt{{\left(x_{Tx}-x_{n,t}\right)}^{2}+{\left(y_{Tx}-y_{n,t}\right)}^{2}}, \end{array}$$

(4) $$\begin{array}{c} \theta_{n,t}\left(x_{n,t}\right)=\arctan\left(\frac{y_{n,t}-y_{Tx}}{x_{n,t}-x_{Tx}}\right), \end{array}$$

where $\left(x_{Tx},y_{Tx}\right)$ denotes the location of the radars, $v_{n,t}$ is a zero-mean white Gaussian noise with covariance matrix $R_{n,t}=\mathrm{diag}\left(\sigma_{r,n}^{2}\left(t\right),\sigma_{\theta ,n}^{2}\left(t\right)\right)$, where $\sigma_{r,n}^{2}\left(t\right)$ and $\sigma_{\theta ,n}^{2}\left(t\right)$ are the variances in the measurement noise on the range and azimuth, respectively, and written as [34],

(5) $$\sigma_{r,n}^{2}\left(t\right)\propto (P_{Tx}|\zeta_{n,t}{{|}^{2}\eta^{2}/{\left(r_{n,t}\left(x_{n,t}\right)\right)}^{4})}^{-1},$$

(6) $$\sigma_{\theta ,n}^{2}\left(t\right)\propto (P_{Tx}|\zeta_{n,t}{{|}^{2}\varsigma^{-2}/{\left(r_{n,t}\left(x_{n,t}\right)\right)}^{4})}^{-1},$$

where ∝ is a proportional operator, $P_{Tx}$ denotes the transmitted power, ς and η denote the 3-dB receive beamwidth and the waveform bandwidth of the phased array radars, $\zeta_{n,t}$ incorporates the effects of phase offsets, and the RCS impact of target *n* on both phase and amplitude.

Note that when a smart target maneuvers, such as sudden changes in velocity or direction, errors arise from the unpredictability of the target’s motion, causing increased measurement errors [35]. In general, the estimation accuracy of the target state can be improved by the adaptive filtering technology at the moment of abrupt maneuvering. It dynamically adjusts the process and measurement noise to accommodate these sudden changes in motion [36,37]. In this paper, the measurement error covariance in dynamic model *m* is defined as $R_{n,t}^{m}$, and $R_{n,t}^{1}<R_{n,t}^{2}<\cdots <R_{n,t}^{M}$, where $R_{n,t}^{1}=\mathrm{diag}\left(\sigma_{r,n}^{2}\left(t\right),\sigma_{\theta ,n}^{2}\left(t\right)\right)$ and < represents that each element in the left matrix is smaller than each corresponding element in the right matrix.

### 2.2. Probabilities of Dynamic Models

After action $a_{n}$ is applied to target *n* at each time, its dynamic model is *m* with probability $u_{n}^{a_{n},m}$, for m=1,2,…,M. Define $U_{n}≜\left[u_{n}^{0},u_{n}^{1}\right]$, where $u_{n}^{a_{n}}={\left[u_{n}^{a_{n},1},u_{n}^{a_{n},2},\ldots ,u_{n}^{a_{n},M}\right]}^{\prime }$. Assume that $u_{n}^{0,1}>u_{n}^{0,m}$, m=2,3,…,M, so targets that are not being tracked are more likely to move into model m=1 (CV). $u_{n}^{1,1}<u_{n}^{1,m}$ for m=2,3,…,M, so targets that are being tracked are less likely to move into model m=1.

Consequently, the smart targets are characterized by the dynamic model probability matrix, and the states x of the targets are affected not only by the CV model but also by the other dynamic models.

### 2.3. TEC State Transition

Denote by ${\hat{x}}_{n,t-1}$ and $P_{n,t-1}$ the dynamic state estimation and TEC state of target *n* at the beginning of time slot t−1. Starting from the initial dynamic state estimation ${\hat{x}}_{n,0}$ and $P_{n,0}$, ${\hat{x}}_{n,t}$, and $P_{n,t}$ are recursively updated over time slots t≥1 being conditioned on the chosen actions, taking into account the dynamic models, as follows.

If the target is tracked at time t−1 ($a_{n,t-1}=1$), then the target will follow the dynamic model probability $u_{n}^{1}$. If it is detected with the detection probability $p_{d}$, the TEC state update follows the non-linear filter as

(7) $$\begin{array}{c} P_{n,t}=\phi_{n}^{1}\left(P_{n,t-1},{\hat{x}}_{n,t-1},z_{n,t},u_{n}^{1}\right). \end{array}$$

For details, the reader is referred to [38].

If the target is not detected, the TEC state of the target can be updated by

(8) $$\begin{array}{c} P_{n,t}=\phi_{n}^{0}\left(P_{n,t-1},u_{n}^{1}\right)=\sum_{m=1}^{M}u_{n}^{1,m}{\bar{P}}_{n,t|t-1}^{m}, \end{array}$$

where

(9) $${\bar{P}}_{n,t|t-1}^{m}=F_{n}^{m}P_{n,t-1}{\left(F_{n}^{m}\right)}^{\prime }+Q_{n}^{m}.$$

If the target is not tracked at time t−1 ($a_{n,t-1}=0$), then

(10) $$\begin{array}{c} P_{n,t}=\phi_{n}^{0}\left(P_{n,t-1},u_{n}^{0}\right)=\sum_{m=1}^{M}u_{n}^{0,m}{\bar{P}}_{n,t|t-1}^{m}. \end{array}$$

Meanwhile, the dynamic state estimation ${\hat{x}}_{n,t}$ can be updated under action 1 or predicted under action 0 based on the dynamic model probability and the non-linear filter.

Note that since the one-step prediction is based on the dynamic model and no measurement is required, the TEC update recursion given by (8) and (10) is deterministic. It follows that action $a_{n,t-1}$ predicts the TEC $P_{n,t}$ through sum-weighted method based on the model probability vector $u_{n}^{a_{n,t-1}}$. When $a_{n,t-1}=1$ but not detected, the target *n* maneuvers with higher probability $u_{n}^{1,m}$, m=2,3,…,M than the maneuvering probability $u_{n}^{0,m}$ under $a_{n,t-1}=0$. Meanwhile, since $q_{n}^{1}<q_{n}^{m}$, m=2,3,…,M, the trace of TEC state $P_{n,t}$ in (8) will be larger than that of (10).

### 2.4. RMABP Model Formulation

The optimal beam scheduling problem for *K* radars and *N* smart targets can be formulated as an infinite-horizon discrete-time RMABP, aiming to achieve the optimal dynamic selection of *K* out of *N* binary-action projects. For such a purpose, a target is identified with a project, which can be operated under the active action (being tracked) and the passive action (being not tracked). The corresponding target’s TEC $P_{n,t}$ and dynamic state estimation ${\hat{x}}_{n,t}$ are taken as the state of each project n=1,2,…,N. $P_{n,t}$ moves over the state space $S_{++}^{L}$ of symmetric positive definite L×L matrices, according to the non-linear filter as shown in Section 2.3. *L* is the dimension of dynamic states. The corresponding ${\hat{x}}_{n,t}\in R^{4}$ can be obtained through the non-linear filter.

For target *n* at time *t*, the immediate cost caused by imposing action $a_{n,t}$ is defined as

(11) $$C_{n}\left(P_{n,t},a_{n,t}\right)≜d_{n}\frac{\mathrm{tr}\left(P_{n,t}\right)}{L}+h_{n}a_{n,t},$$

where tr(·) denotes the trace of a matrix, $d_{n}>0$ is the target’s weight, which is used to model the importance of targets, and $h_{n}\geq 0$ is a measurement cost.

The radar network selects at most *K* out of *N* smart targets to track in each time slot *t*. Each radar can only steer one beam to track one target at each time, which is formulated as the constraint

(12) $$\sum_{n=1}^{N}a_{n,t}\leq K,\;t=0,1,2,\ldots .$$

Given the joint model probabilities $U={\left(U_{n}\right)}_{n=1}^{N}$, joint TEC state at the beginning $P_{0}={\left(P_{n,0}\right)}_{n=1}^{N}$, and joint dynamic state estimation at the beginning $X_{0}={\left({\hat{x}}_{n,0}\right)}_{n=1}^{N}$, the dynamic optimization problem with discounted cost over an infinite time horizon is formulated as follows:

(13) $$\begin{array}{cc} \min_{\pi \in \Pi (K)} & E_{P_{0},X_{0}}^{\pi }\left[\sum_{t=0}^{\infty }\sum_{n=1}^{N}\beta^{t}C_{n}\left(P_{n,t},a_{n,t}\right)\right],s.t.:\;\left(12\right). \end{array}$$

where 0<β<1 is the discount factor, $E_{P_{0},X_{0}}^{\pi }\left[\cdot \right]$ is expectation under policy π conditioned on the initial state $P_{0}$, $X_{0}$, process noise and measurement noise, and denote by Π(K) the set of stationary scheduling policies that satisfy (12). $V^{*}\left(P_{0},X_{0}\right)$ denotes the optimal cost of problem (13).

## 3. RMABP-Based Beam Scheduling Policy

Generally, determining an optimal policy for problem (13) is a computationally intractable task, due both to the curse of dimensionality and to the continuous state space. This section next discusses how Whittle’s approach [16] for obtaining an unscented sampling-based MP (US-MP) index policy would apply to this smart-target tracking problem and discusses the challenges it poses. See also [29].

### 3.1. Problem Relaxation and Decomposition

The sample-path constraint (12) over the infinite-horizon time can be formulated as the expected total discounted constraint,

(14) $$E_{P_{0},X_{0}}^{\pi }\left[\sum_{t=0}^{\infty }\sum_{n=1}^{N}\beta^{t}a_{n,t}\right]\leq \frac{K}{1-\beta },$$

which only requires that the expected total discounted number of selected targets does not exceed K/(1−β).

Now, consistently with the above notation Π(K), let Π(N) represent the set of stationary scheduling policies capable of activating any quantity of projects (tracking any number of targets) at each time. Then, a relaxation of problem (13) is obtained as follows.

(15) $$\begin{array}{cc} \min_{\pi \in \Pi (N)}\; & E_{P_{0},X_{0}}^{\pi }\left[\sum_{t=0}^{\infty }\sum_{n=1}^{N}\beta^{t}C_{n}\left(P_{n,t},a_{n,t}\right)\right],s.t.:\;\left(14\right). \end{array}$$

Therefore, the minimum cost $V^{R}\left(P_{0},X_{0}\right)$ derived from problem (15) establishes a lower bound on $V^{*}\left(P_{0},X_{0}\right)$.

Attach now a Lagrange multiplier λ≥0 to the aggregate constraint (14). The Lagrangian relaxation applied to (15) is

(16) $$\begin{array}{c} \min_{\pi \in \Pi (N)}E_{P_{0},X_{0}}^{\pi }\left[\sum_{t=0}^{\infty }\sum_{n=1}^{N}\beta^{t}\left\{C_{n}\left(P_{n,t},a_{n,t}\right)+\lambda a_{n,t}\right\}\right]-\frac{K\lambda }{1-\beta }. \end{array}$$

Given a λ≥0, the optimal value of the Lagrangian relaxation, denoted as $V^{L}\left(P_{0},X_{0};\lambda \right)$, serves as a lower bound for $V^{R}\left(P_{0},X_{0}\right)$.

By searching for an optimal Lagrange multiplier $\lambda^{*}\left(P_{0},X_{0}\right)$ based on the Lagrangian relaxation, the best lower bound for $V^{R}\left(P_{0},X_{0}\right)$ is obtained. This defines the dual problem of (15):

(17) $$V^{D}\left(P_{0},X_{0}\right)≜\max_{\lambda \geq 0}\;V^{L}\left(P_{0},X_{0};\lambda \right).$$

Problem (16) can be decomposed into *N* single-target independent subproblems given by

(18) $$\min_{\pi^{n}\in \Pi^{n}}E_{P_{n,0},{\hat{x}}_{n,0}}^{\pi^{n}}\left[\sum_{t=0}^{\infty }\beta^{t}\left\{C_{n}\left(P_{n,t},a_{n,t}\right)+\lambda a_{n,t}\right\}\right].$$

In (18), $\Pi^{n}$ is the set of stationary scheduling policies corresponding to target *n*. Here, multiplier λ has the economic interpretation of an additional tracking cost.

### 3.2. Unscented Sampling-Based TEC Update

Note that the TEC state update is related to the dynamic state and the measurement at the next time in (7). However, the target future dynamic state and measurement of multiple steps cannot be known at the current time. In the multi-step action-making for Problem (18), this paper predicts them based on the US method, which has a lower computational burden than the Monte Carlo sampling method.

The US method generates the 2L+1 Sigma points $\epsilon_{n,t-1}^{i}$ to sample the measurements. This allows for updating the TEC states and dynamic states through a non-linear filter without actual measurements. The Sigma points and weights $\alpha_{n}^{i}$ are

(19) $$\epsilon_{n,t-1}^{i}=\left\{\begin{array}{cc} {\hat{x}}_{n,t-1}, & i=0\\ {\hat{x}}_{n,t-1}+\sqrt{\left(L+\xi \right)}\rho_{i}, & i=1,\ldots ,L\\ {\hat{x}}_{n,t-1}-\sqrt{\left(L+\xi \right)}\rho_{i}, & i=L+1,\ldots ,2L \end{array}\right.$$

(20) $$\alpha_{n}^{i}=\left\{\begin{array}{cc} \frac{\xi }{L+\xi }, & i=0\\ \frac{\xi }{2(L+\xi )}, & i=1,2,\ldots ,2L \end{array}\right.$$

where $\rho_{i}$ denotes the *i*-th row of the matrix after Cholesky decomposition of $P_{n,t-1}$, $\xi =\gamma^{2}\left(L+\kappa \right)-L$ is the scale parameter, γ represents the distribution parameter that determines the distribution degree of Sigma points, and  κ generally takes the value of 3−L.

Based on the Sigma points $\epsilon_{n,t-1}^{i}$, the dynamic states and corresponding measurements of diverse dynamic models can be sampled according to

(21) $${\bar{x}}_{n,t}^{i,m}=F_{n}^{m}\epsilon_{n,t-1}^{i}+w_{n,t-1}^{m},$$

(22) $$z_{n,t}^{i,m}=H_{n}\left({\bar{x}}_{n,t}^{i,m}\right)+v_{n,t}.$$

Through the non-linear filter, the Sigma point $\epsilon_{n,t-1}^{i}$ can be updated with the measurement $z_{n,t}^{i,m}$. For details, the reader is referred to [38]. Then, the state estimation ${\hat{x}}_{n,t}^{i,m}$ and TEC estimation ${\hat{P}}_{n,t}^{i,m}$ are obtained.

The dynamic state and TEC are updated by

(23) $${\hat{x}}_{n,t}=\sum_{m=1}^{M}u_{n}^{1,m}\sum_{i=0}^{2L}\alpha_{n}^{i}{\hat{x}}_{n,t}^{i,m},$$

(24) $$P_{n,t}=\sum_{m=1}^{M}u_{n}^{1,m}\sum_{i=0}^{2L}\alpha_{n}^{i}{\hat{P}}_{n,t}^{i,m}.$$

In the subsequent Whittle index policy, when the tracking action is performed and the target is successfully detected, the future TEC update is derived as

(25) $$\begin{array}{c} P_{n,t}=\psi_{n}^{1}\left(P_{n,t-1},{\hat{x}}_{n,t-1},u_{n}^{1}\right)=\sum_{m=1}^{M}u_{n}^{1,m}\sum_{i=0}^{2L}\alpha_{n}^{i}{\hat{P}}_{n,t}^{i,m}. \end{array}$$

Note that (25) is only used in potential future multi-step action-making, such as the calculation of the Whittle index. Additionally, (7) is used in TEC transitions when performing online scheduling.

### 3.3. Computing the Lower Bound $V^{D}\left(P_{0},X_{0}\right)$

In the one-dimensional case with scalar state space for a single bandit process, scalar TEV state updates are deterministic. The lower bound $V^{D}\left({\left(P_{n,0}\right)}_{n=1}^{N}\right)$ is defined without dynamic state estimations ${\left({\hat{x}}_{n,0}\right)}_{n=1}^{N}$. $V^{D}\left({\left(P_{n,0}\right)}_{n=1}^{N}\right)$ can be calculated through the value iteration approach [39], where $P_{n,0}$ is the initial TEV state of target *n*.

In the multi-dimensional case, consider the lower bound $V^{D}\left(P_{0},X_{0}\right)$ in (17), which is the optimal value of the Lagrangian dual problem. Based on the Bellman equation, the optimal value function is firstly derived for the processes $\left\{P_{n,t},t=0,1,\ldots \right\}$ and $\left\{{\hat{x}}_{n,t},t=0,1,\ldots \right\}$ of target *n* in (18). Given the Lagrange multiplier λ, the optimal value function $v_{n}^{\lambda ,*}\left(P_{n,t},{\hat{x}}_{n,t}\right)$ with the detection probability $p_{d}$ is

(26) $$\begin{array}{cc} v_{n}^{\lambda ,*}\left(P_{n,t},{\hat{x}}_{n,t}\right)=\min_{a_{n,t}\in \left\{0,1\right\}} & \{C_{n}\left(P_{n,t},a_{n,t}\right)+\lambda a_{n,t}+\beta E\left[I_{a_{n,t}=0}\;v_{n}^{\lambda ,*}\left(\phi_{n}^{0}\left(P_{n,t},u_{n}^{0}\right),{\hat{x}}_{n,t+1}\right)\right]\\ \; & +\beta E[I_{a_{n,t}=1}[p_{d}v_{n}^{\lambda ,*}\left(\psi_{n}^{1}\left(P_{n,t},{\hat{x}}_{n,t},u_{n}^{1}\right),{\hat{x}}_{n,t+1}\right)\\ \; & +\left(1-p_{d}\right)v_{n}^{\lambda ,*}\left(\phi_{n}^{0}\left(P_{n,t},u_{n}^{1}\right),{\hat{x}}_{n,t+1}\right)]]\}. \end{array}$$

Subsequently, given the Lagrange multiplier λ in (16), the optimal value of the Lagrangian relaxation $V^{L}\left(P_{0},X_{0};\lambda \right)$ can be derived by substituting (26) into it, as given by

(27) $$\begin{array}{c} V^{L}\left(P_{0},X_{0};\lambda \right)=\sum_{n=1}^{N}v_{n}^{\lambda ,*}\left(P_{n,0},{\hat{x}}_{n,0}\right)-\frac{K\lambda }{1-\beta }. \end{array}$$

Based on the established value function and a given λ, the *k*-th iteration of value function $v_{n,k}^{\lambda }\left(P_{n,t},{\hat{x}}_{n,t}\right)$ is defined as

(28) $$\begin{array}{cc} v_{n,k}^{\lambda }\left(P_{n,t},{\hat{x}}_{n,t}\right)=\min_{a_{n,t}\in \left\{0,1\right\}} & \{C_{n}\left(P_{n,t},a_{n,t}\right)+\lambda a_{n,t}+\beta E\left[I_{a_{n,t}=0}v_{n,k-1}^{\lambda }\left(\phi_{n}^{0}\left(P_{n,t},u_{n}^{0}\right),{\hat{x}}_{n,t+1}\right)\right]\\ \; & +\beta E[I_{a_{n,t}=1}[p_{d}v_{n,k-1}^{\lambda }\left(\psi_{n}^{1}\left(P_{n,t},{\hat{x}}_{n,t},u_{n}^{1}\right),{\hat{x}}_{n,t+1}\right)\\ \; & +\left(1-p_{d}\right)v_{n,k-1}^{\lambda }\left(\phi_{n}^{0}\left(P_{n,t},u_{n}^{1}\right),{\hat{x}}_{n,t+1}\right)]]\}. \end{array}$$

Then, the value iteration approach continues the iterative process over the state space until convergence of values on diverse joint states $P_{n,t}$ and ${\hat{x}}_{n,t}$. Finally, the optimal value $v_{n}^{\lambda ,*}\left(P_{n,t},{\hat{x}}_{n,t}\right)$ in (26) is obtained. Given the initial joint states $P_{0}$, $X_{0}$ and a given λ, the minimum cost $V^{L}\left(P_{0},X_{0};\lambda \right)$ can be computed by (27). The lower bound $V^{D}\left(P_{0},X_{0}\right)$ of the dual problem in (17) can be computed by searching a λ∈[0,∞) to satisfy (17).

Above all, this paper regards the lower bound of problem (17) as the optimal cost of the original problem (13) to further compare discounted cost per target performances of policies, given a constant scale ratio K/N.

### 3.4. Indexability and Whittle Index Policy

Consider now the following structural property of subproblems (18), referred to as indexability in [16], which is formulated next as in (Section 3.2 in [30]). Note that target *n*’s subproblem (18) is indexable if there exists an index $\lambda_{n}^{*}:\left(S_{++}^{L},R^{4}\right)\to R$ that characterizes its optimal policies, as follows: for any λ∈R, when the target is in the TEC state $P_{n}$ and dynamic state ${\hat{x}}_{n}$, $\lambda_{n}^{*}\left(P_{n},{\hat{x}}_{n}\right)>\lambda$ is a necessary and sufficient condition so that taking action $a_{n}=1$ is optimal. Conversely, if $\lambda_{n}^{*}\left(P_{n},{\hat{x}}_{n}\right)\leq \lambda$, then the optimal action is $a_{n}=0$.

If each subproblem were indexable, the Whittle index policy would be to track up to *K* out of the *N* targets with largest index values, among those with non-negative indices. If the current index of a target is negative, it is not tracked.

Yet, at present, it is unknown whether restless projects with the multi-dimensional non-linear filter such as those above are indexable, even for a single dynamic model (M=1). Indexability has only been established (in [40]) for the special case of target tracking with scalar Kalman filter dynamic and a single dynamic model, by applying the PCL-indexability approach for scalar-state projects in [22].

### 3.5. PCL-Indexability Approach for Scalar-State Targets

This subsection next outlines the PCL-indexability approach for scalar-state restless bandit projects as it applies to the present model (so L=1). Accordingly, the dynamic and measurement models are one-dimensional and linear. The TEV state $P_{n,t}$ transitions are redefined as

(29) $$P_{n,t}=\left\{\begin{array}{cc} \mu_{n}^{1}\left(P_{n,t-1},u_{n}^{1}\right), & a_{n,t-1}=1,\;\mathrm{detected}\\ \mu_{n}^{0}\left(P_{n,t-1},u_{n}^{1}\right), & a_{n,t-1}=1,\;\mathrm{not}\;\mathrm{detected}\\ \mu_{n}^{0}\left(P_{n,t-1},u_{n}^{0}\right), & a_{n,t-1}=0 \end{array}\right.$$

where

(30) $$\mu_{n}^{1}\left(P_{n,t-1},u_{n}^{1}\right)=\sum_{m=1}^{M}u_{n}^{1,m}{\hat{P}}_{n,t}^{m},$$

(31) $$\mu_{n}^{0}\left(P_{n,t-1},u_{n}^{1}\right)=\sum_{m=1}^{M}u_{n}^{1,m}{\bar{P}}_{n,t|t-1}^{m},$$

(32) $$\mu_{n}^{0}\left(P_{n,t-1},u_{n}^{0}\right)=\sum_{m=1}^{M}u_{n}^{0,m}{\bar{P}}_{n,t|t-1}^{m},$$

(33) $${\hat{P}}_{n,t}^{m}=\left(1-K_{n,t}^{m}H_{n}\right){\bar{P}}_{n,t|t-1}^{m},$$

(34) $$K_{n,t}^{m}={\bar{P}}_{n,t|t-1}^{m}H_{n}^{\prime }{\left(H_{n}{\bar{P}}_{n,t|t-1}^{m}H_{n}^{\prime }+R_{n}^{m}\right)}^{-1},$$

(35) $${\bar{P}}_{n,t|t-1}^{m}=F_{n}^{m}P_{n,t-1}{\left(F_{n}^{m}\right)}^{\prime }+Q_{n}^{m}.$$

And $F_{n}^{m}$, $Q_{n}^{m}$, and $R_{n}^{m}$ denote the TEV state transition ratio, process noise variance, and measurement noise variance of dynamic model *m*, respectively.

In the sequel, this subsection pays attention to the MP index of a single target with multiple dynamic models and the detection probability $p_{d}$ as described above. This subsection drops the subscript *n* from notations.

Starting from an initial TEV state $P_{0}=P\in S_{++}≜\left(0,\infty \right)$, for a policy π, define the cost metric and work metric

(36) $$F\left(P,\pi \right)≜E_{P}^{\pi }\left[\sum_{t=0}^{\infty }\beta^{t}C\left(P_{t},a_{t}\right)\right],$$

(37) $$G\left(P,\pi \right)≜E_{P}^{\pi }\left[\sum_{t=0}^{\infty }\beta^{t}a_{t}\right].$$

The subproblem (18) can be reformulated in scalar TEV states as

(38) $$\min_{\pi \in \Pi }\;F\left(P,\pi \right)+\lambda G\left(P,\pi \right).$$

The indexability of subproblem (38) is studied under deterministic stationary policies and threshold policies. Deterministic stationary policies are represented as (Borel measurable) subsets of TEV states, where the corresponding target is tracked. An active set denoted as $B\subseteq S_{++}$ is employed to characterize the *B*-active policy. In particular, for any given threshold level $Z\in \bar{S}≜S_{++}\cup \left\{-\infty ,\infty \right\}$, this is designated as the *Z*-threshold policy. This means that, for a target in TEV state *P*, if P>Z, the target is tracked; otherwise, it is not tracked. Therefore, a *Z*-threshold policy has active set $B\left(Z\right)≜\left\{P\in S_{++}:P>Z\right\}$. It is clear that, if 0<Z<∞, then B(Z)=(Z,∞); if Z≤0, then $B\left(Z\right)=S_{++}$; and if Z=∞, then B(Z)=∅. Denote the corresponding cost and work metrics in (36) and (37) by F(P,Z) and G(P,Z), respectively.

Given a threshold *Z*, the cost and work metrics are characterized by the functional equations.

(39) $$F\left(P,Z\right)=\left\{\begin{array}{cc} C\left(P,1\right)+\beta p_{d}F\left(\mu^{1}\left(P,u^{1}\right),Z\right)+\beta \left(1-p_{d}\right)F\left(\mu^{0}\left(P,u^{1}\right),Z\right), & P>Z\\ C\left(P,0\right)+\beta F\left(\mu^{0}\left(P,u^{0}\right),Z\right), & P\leq Z. \end{array}\right.$$

and

(40) $$G\left(P,Z\right)=\left\{\begin{array}{cc} 1+\beta p_{d}G\left(\mu^{1}\left(P,u^{1}\right),Z\right)+\beta \left(1-p_{d}\right)G\left(\mu^{0}\left(P,u^{1}\right),Z\right), & P>Z\\ \beta G\left(\mu^{0}\left(P,u^{0}\right),Z\right), & P\leq Z. \end{array}\right.$$

In practice, such metrics can be approximately computed by a value-iteration scheme with a finite truncated time horizon τ, as discussed in (Section 11 in [22]).

Next, 〈a,Z〉 represents the policy that executes action *a* at time t=0 and subsequently follows the *Z*-threshold policy from t=1 onwards in (39) and (40). Accordingly, the corresponding marginal metrics for the previously discussed measures are defined specifically: marginal cost metric f(P,Z)≜F(P,〈0,Z〉)−F(P,〈1,Z〉) and the marginal work metric g(P,Z)≜G(P,〈1,Z〉)−G(P,〈0,Z〉). If g(P,Z)>0, the MP metric function can be further defined by

(41) $$\mathrm{mp}\left(P,Z\right)=\frac{f(P,Z)}{g(P,Z)}.$$

If g(P,P)>0 for all *P*, the *MP index* is defined by

(42) $${\mathrm{mp}}^{*}\left(P\right)≜\mathrm{mp}\left(P,P\right).$$

As in (Definition 7 in [22]), the subproblem (38) is PCL-indexable (with respect to threshold policies) if the following PCL-indexability conditions hold:

- (PCLI1) g(P,Z)>0 for every state *P* and threshold *Z*;
- (PCLI2) ${\mathrm{mp}}^{*}\left(P\right)$ is monotone non-decreasing, continuous, and bounded below;
- (PCLI3) for each *P*, the metrics F(P,Z), G(P,Z), and the index ${\mathrm{mp}}^{*}\left(P\right)$ are related by: for $-\infty <Z_{1}<Z_{2}<\infty$,
  $$F\left(P,Z_{2}\right)-F\left(P,Z_{1}\right)=\int_{\left(Z_{1},Z_{2}\right]}{\mathrm{mp}}^{*}\left(Z\right)\;G\left(P,dZ\right),$$
  where the right-hand side is a Lebesgue–Stieltjes integral.

The interest of the above PCL-indexability conditions lies in their applicability through the verification theorem in (Theorem 1 in [22]), which ensures that, for a scalar-state project, conditions (PCLI1)–(PCLI3) above imply that the project is indexable, and the MP index ${\mathrm{mp}}^{*}\left(P\right)$ is its Whittle index. Ref. [40] and Theorem 1 in [22] provide the following Lemma 1 to prove indexability for the special case of a single dynamic model (M=1).

**Lemma 1.**  If subproblem (38) is PCL-indexable, then it is indexable with Whittle index ${\mathrm{mp}}^{*}\left(P\right)$.

This paper conjectures that the model satisfies such conditions, and will present partial numerical evidence supporting satisfaction of such conditions.

### 3.6. A US-Based MP Index for Multi-Dimensional State Targets

In the case of target dynamic states moving over in an *L*-dimensional state space $R^{L}$, with L≥2, the TEC matrix indicates the tracking accuracy of the target state’s components, e.g., location and velocity. Since the TEC state is an L×L matrix, a convenient scalar measure of tracking performance is given by its trace. This section leverages the past success of the PCL-indexability and extends a new heuristic approach to define a US-MP index for the multi-dimensional case.

In particular, the cost metric $F(P,\hat{x},\pi )$ and the work metric $G(P,\hat{x},\pi )$ are defined by replacing the one-dimensional *P* with the TEC P and the dynamic state $\hat{x}$ in (36) and (37). The target’s optimal tracking subproblem (18) becomes

(43) $$\min_{\pi \in \Pi }\;F\left(P,\hat{x},\pi \right)+\lambda G\left(P,\hat{x},\pi \right).$$

The meaning of threshold policies is defined next in the present setting. For such a purpose, the *Z*-threshold policy tracks the target in states P and $\hat{x}$ if and only if tr(P)/L>Z. Then, the corresponding cost metric $F(P,\hat{x},Z)$ and work metric $G(P,\hat{x},Z)$ are the following functional equations:

(44) $$\begin{array}{c} F\left(P,\hat{x},Z\right)=\left\{\begin{array}{cc} C\left(P,1\right)+\beta p_{d}F\left(\psi^{1}\left(P,\hat{x},u^{1}\right),{\hat{x}}^{*},Z\right)\\ \;\;\;\;\;\;\;\;\;\;\;+\beta \left(1-p_{d}\right)F\left(\phi^{0}\left(P,u^{1}\right),{\hat{x}}^{*},Z\right), & \mathrm{tr}(P)/L>Z\\ C\left(P,0\right)+\beta F(\phi^{0}\left(P,u^{0}\right),{\hat{x}}^{*},Z), & \mathrm{tr}(P)/L\leq Z. \end{array}\right. \end{array}$$

(45) $$\begin{array}{c} G\left(P,\hat{x},Z\right)=\left\{\begin{array}{cc} 1+\beta p_{d}G\left(\psi^{1}\left(P,\hat{x},u^{1}\right),{\hat{x}}^{*},Z\right)\\ \;\;\;+\beta \left(1-p_{d}\right)G\left(\phi^{0}\left(P,u^{1}\right),{\hat{x}}^{*},Z\right), & \mathrm{tr}(P)/L>Z\\ \beta G(\phi^{0}\left(P,u^{0}\right),{\hat{x}}^{*},Z), & \mathrm{tr}(P)/L\leq Z. \end{array}\right. \end{array}$$

where ${\hat{x}}^{*}$ denotes the subsequent dynamic state. Again, such solutions can be approximated through a value-iteration approach using a truncated time horizon τ.

Now, similar to the scalar case above, the marginal cost metric $f(P,\hat{x},Z)$ and the marginal work metric $g(P,\hat{x},Z)$ are defined through the 〈a,Z〉 method in (44) and (45). Then, the US-MP metric is defined by

(46) $$\begin{array}{cc} \mathrm{mp}(P,\hat{x},Z) & =\frac{f(P,\hat{x},Z)}{g(P,\hat{x},Z)}\\ \; & =\frac{F\left(P,\hat{x},\langle 0,Z\rangle \right)-F\left(P,\hat{x},\langle 1,Z\rangle \right)}{G\left(P,\hat{x},\langle 1,Z\rangle \right)-G\left(P,\hat{x},\langle 0,Z\rangle \right)}, \end{array}$$

provided that $g(P,\hat{x},Z)>0$.

The corresponding US-MP index is defined by

(47) $${\mathrm{mp}}^{*}\left(P,\hat{x}\right)≜\mathrm{mp}\left(P,\hat{x},\mathrm{tr}\left(P\right)/L\right),$$

provided that $g(P,\hat{x},\mathrm{tr}\left(P\right)/L)>0$ for all P.

The cost metric is defined by the trace of TEC states in (11), and tr(P)/L is considered in the Z-threshold policy in (44) and (45). In particular, the determinant metric of TEC states can also be considered, provided these metric settings are consistent.

However, there is currently no theory supporting any relation of the index ${\mathrm{mp}}^{*}\left(P,\hat{x}\right)$ with the Whittle index in the multi-dimensional case, assuming that both are well defined.

### 3.7. Scheduling Scheme Based on the MP Index Policy

Section 3.5 and Section 3.6 discussed the MP-based indices for a bandit process with scalar and multi-dimensional state spaces, respectively. In general, the MP-based index policy prioritizes tracking specific targets based on their state-dependent MP indices, regardless of the dimensions of the state, in descending order. At each time *t*, the *K* targets are selected with the largest indexes, and the action $a_{n,t}$, n=1,…,N, is generated. Then the TEC and dynamic states of *N* targets are updated depending on actions and a non-linear filter. Finally, calculate the cost in time *t*. The pseudo-code of implementing the MP index policy with given indices is provided in Algorithm 1, which is applicable to both cases with one-dimensional and multi-dimensional state spaces.   
**Algorithm 1:** The scheduling scheme based on the MP index policy
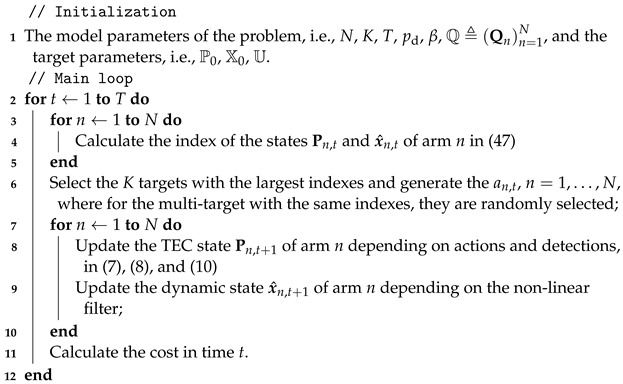


In the Whittle index policy, for the case with scalar bandit states, if the actions of each target across τ time horizon are all assumed as 1, the computational complexity of (42) is $O(N2^{\tau +1})$. Note that due to the combination of actions, i.e., 0 and 1, across τ time horizon, the computational complexity of the Whittle index is less than $O(N2^{\tau +1})$. For the case with multi-dimensional state space for each bandit process, the truncated time horizon τ is the recursive horizon in (44) and (45). In general, the computational complexity of the non-linear filter is $O(L^{2})$. The complexity for computing the indices in (47) is $O(N2^{\tau +1}L^{2})$.

For non-predetermined complex dynamic models, data-driven approaches can learn the dynamic features from the tracking data. Based on this learned information, the proposed policy can introduce new dynamic models and modify the model probability matrix U. Meanwhile, the adaptive filtering technology helps prevent tracking performance degradation during the learning process [36,37].

## 4. Simulation and Analysis

This section reports on numerical experiments to assess the satisfaction of the PCL-indexability conditions and the effectiveness of the proposed Whittle (MP) index policy in the scalar-state case.

The proposed US-MP index policy will be compared with the two myopic baselines, i.e., the TEC index policy and the myopic policy, in the multi-dimensional state cases. Since the dynamic state $x_{n,t}$ belongs to $R^{4}$, we conclude that L=4. Based on [29], in the multi-dimensional state case, the *TEC index* policy focuses on the current cost and defines the index by

(48) $$\lambda^{\mathrm{TEC}}\left(P\right)=d\frac{\mathrm{tr}(P)}{L}.$$

The TEC index represents the magnitude of the current covariance in each target. The computational complexity of the TEC index derivation is O(NL).

The myopic policy focuses on higher-cost targets at the immediate subsequent moment and calculates the one-step prediction cost under the tracking action 1 and the detection probability $p_{d}$, with the indices being given by

(49) $$\begin{array}{c} \lambda^{\mathrm{myopic}}=\frac{d\left[p_{d}\mathrm{tr}\left(\psi^{1}\left(P,\hat{x},u^{1}\right)\right)+\left(1-p_{d}\right)\mathrm{tr}\left(\phi^{0}\left(P,u^{1}\right)\right)\right]}{L}. \end{array}$$

The computational complexity for $\lambda^{\mathrm{myopic}}$ is $O(2NL^{2})$. The baseline policies exhibit lower computational complexity but, in general, fail to take into consideration the long-term effects of the employed actions.

The performance metrics include the discounted cost in (13) and weighted RMSE (WRMSE), which is defined in (50).

(50) $$\begin{array}{c} {\mathrm{WRMSE}}_{t}≜\sqrt{\frac{1}{N_{\mathrm{mc}}}\sum_{m_{c}=1}^{N_{\mathrm{mc}}}\sum_{n=1}^{N}\frac{d_{n}}{\left|d\right|}\left[{\left({\hat{x}}_{n,t}\left(m_{c}\right)-x_{n,t}\right)}^{2}+{\left({\hat{y}}_{n,t}\left(m_{c}\right)-y_{n,t}\right)}^{2}\right]}, \end{array}$$

where $\left({\hat{x}}_{n,t}\left(m_{c}\right),{\hat{y}}_{n,t}\left(m_{c}\right)\right)$ is the estimated location of target *n* in $m_{c}$-th Monte-Carlo simulation. $N_{\mathrm{mc}}$ is the total number of Monte-Carlo trials, $d≜(d_{1},\ldots ,d_{N})$ and |d| denotes the sum of weights. Then, the average WRMSE (AWRMSE) is defined as

(51) $$\begin{array}{c} \mathrm{AWRMSE}≜\frac{1}{T}\sum_{t=1}^{T}{\mathrm{WRMSE}}_{t}. \end{array}$$

### 4.1. PCL-Indexability Conditions and MP Index Evaluation for Scalar-States

Consider the PCL-indexability conditions stated in Section 3.5 as they apply to scalar-state targets. A sample of numerical evidence is presented below to support the conjecture that the model satisfies such conditions.

To numerically test the conditions, consider the targets have M=2 dynamic models, which are referred to as CV (m=1) and CT (m=2). Set up the parameter values as follows. $F^{1}=1.1$, $F^{2}=1.7$, $Q^{1}=10$, $Q^{2}=20>Q^{1}$, H=1, $R^{1}=20$, and $R^{2}=80$. $F^{2}=1.7$ represents a greater kinematic maneuverability of the CT dynamic model, which has a higher measurement noise variance $R^{2}=80$. The detection probability $p_{d}$ is 0.8. There are no measurement costs (h=0). Consider two types of smart targets, called reckless and cautious, where reckless targets have a higher probability of maneuvering (using the CT model) when tracked than cautious targets. Hence, the dynamic model probability matrix $U=\left[u^{0},u^{1}\right]$ differs with the target types. Specifically, this subsection selects an arbitrary parameter instance to conduct the simulation experiment.

(52) $$U=\left\{\begin{array}{cc} \left[{\left[0.90,0.10\right]}^{\prime },{\left[0.20,0.80\right]}^{\prime }\right], & \mathrm{for}\;\mathrm{reckless}\;\mathrm{targets}\\ \left[{\left[0.95,0.05\right]}^{\prime },{\left[0.60,0.40\right]}^{\prime }\right], & \mathrm{for}\;\mathrm{cautious}\;\mathrm{targets}. \end{array}\right.$$

where, if target $n_{1}$ belongs to reckless targets, target $n_{2}$ belongs to cautious targets, then $u_{n_{1}}^{0,m}>u_{n_{2}}^{0,m}$ and $u_{n_{1}}^{1,m}>u_{n_{2}}^{1,m}$, m=2,…,M. It is noted that the TEC states are linearly related to the matrix U, and the absolute values of the indices vary as U takes different values.

In the following numerical experiments, the infinite series defining the metrics of interest have been approximately computed by using the truncated time horizon τ=16. The discount factor is β=0.75 and the weight is d=1.

**Figure 2:**
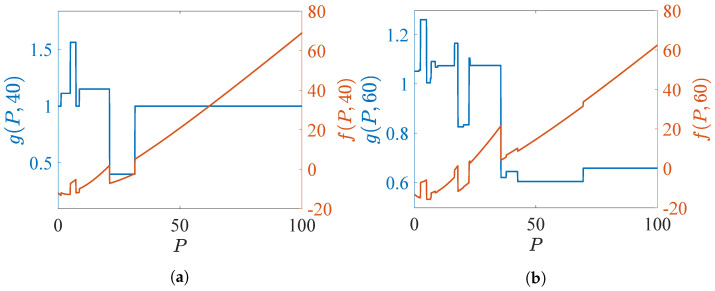
Marginal work metric g(P,Z) and marginal cost metric f(P,Z) for reckless targets. (**a**) Z=40 (**b**) Z=60.

**Figure 3:**
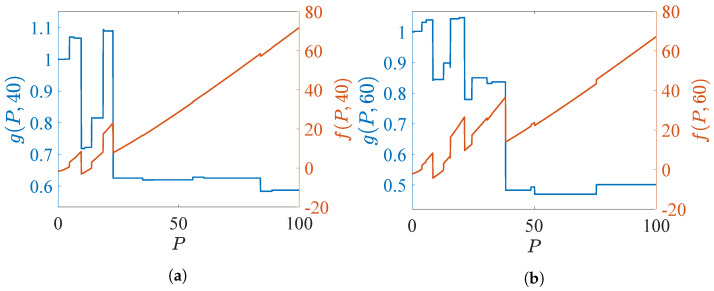
Marginal work metric g(P,Z) and marginal cost metric f(P,Z) for cautious targets. (**a**) Z=40 (**b**) Z=60.

Start with PCL-indexability condition (PCLI1), namely that marginal work metric g(P,Z) be positive for each TEV state *P* and threshold *Z*. Figure 2 and Figure 3 plot both g(P,Z) and f(P,Z) against *P* for several choices of threshold *Z*, for reckless targets and cautious targets, respectively. The plots support the validity of condition (PCLI1), as g(P,Z)>0 in each instance. Results that have been observed under other parameter choices are not reported here.

**Figure 4:**
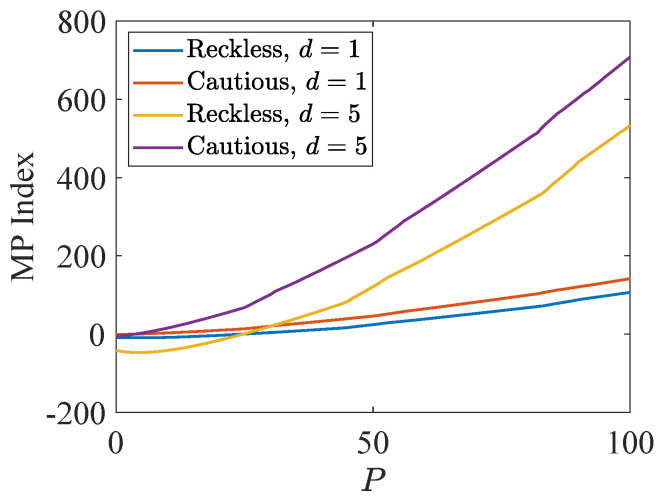
MP indices for the two target types.

Regarding PCL-indexability condition (PCLI2), namely that the MP index defined by ${\mathrm{mp}}^{*}\left(P\right)≜f\left(P,P\right)/g\left(P,P\right)$ be bounded below, continuous and monotone non-decreasing, Figure 4 plots the MP index for the two target types and the two weights d=1,5. The plot is clearly consistent with condition (PCLI2), and results have been observed in other instances not reported here. Due to the higher probability of a cautious target acquiring a lower cost with the same state *P* under action 1, cautious targets obtain a higher index value than reckless targets in all states, which indicates that the MP index policy takes the tracking action prudently for reckless targets.

In summary, the above partial numerical evidence shows that the model of concern satisfies PCL-indexability conditions (PCLI1) and (PCLI2), and hence the MP index shall be further used as a surrogate of the Whittle index and referred to as the Whittle index.

### 4.2. Performance Results with Scalar States

In this subsection, N=8 smart targets are tracked by the radars network with K=1,2,3 radars. For each target, the radar tracking time horizon T=40 s and β=0.75. The Whittle index policy is set with the truncated time τ=16; The TEV index policy defines the index $\lambda^{\mathrm{TEV}}\left(P\right)=d\cdot P$; And the myopic policy that degenerates to $\lambda^{\mathrm{myopic}}=d\cdot \left[p_{d}\mu^{1}\left(P,u^{1}\right)+\left(1-p_{d}\right)\mu^{0}\left(P,u^{1}\right)\right]$ to assess tracking performances.

The parameters $F_{n}^{1}$, $F_{n}^{2}$, $Q_{n}^{1}$, $Q_{n}^{2}$, $R_{n}^{1}$, $R_{n}^{2}$, and $h_{n}$ of target *n* and *H* are assumed to be the same as Section 4.1, n=1,2,…,N. The initial dynamic state $x_{n,0}$ and TEV state $P_{n,0}$ follow U(0,50) and U(0,40), respectively. Then, three scenarios are defined with only reckless type, cautious type, and each type of target accounting for 4, respectively. Set $d_{n}=5$, n=1,…,K, and $d_{n}=1$, n=K+1,…,N in the three scenarios.

Table 1 shows the discounted costs and AWRMSEs obtained by the three policies. Each policy is analyzed by $N_{\mathrm{mc}}=1000$ Monte Carlo simulations. Meanwhile, the percentages in parentheses indicate the metric value ratio of the corresponding policy over the myopic policy. The Whittle index policy can obtain the best scheduling performance and outperform the TEV index and myopic policies. The myopic policy focuses on minimizing the one-step cost under the detection probability $p_{d}$, but does not consider multi-step decision making. As a result, it tends to favor conservative actions and fails to outperform the TEV policy. Consequently, the performance of the TEV policy is better than the myopic policy.

**Figure 5:**
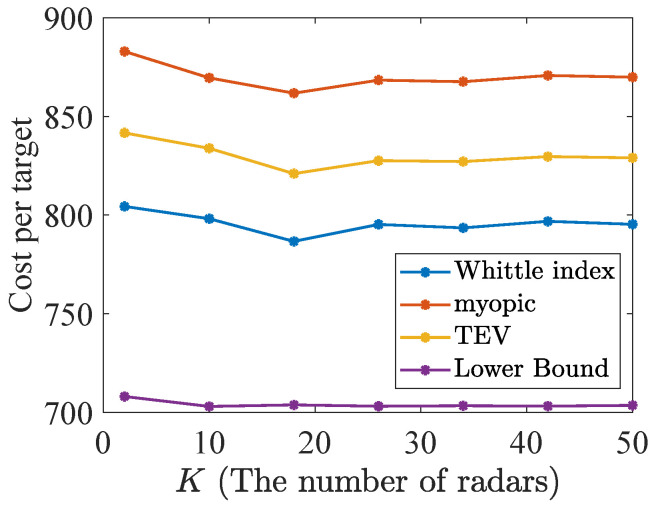
The discounted cost per target in the constant ratio 1/4.

Additionally, this subsection validates the near optimality of the Whittle index policy discussed in Section 3.4 through 1000 Monte Carlo simulations, when the number of radars and targets increases with a fixed ratio K/N=1/4. The parameters $F_{n}^{1}$, $F_{n}^{2}$, $Q_{n}^{1}$, $Q_{n}^{2}$, $R_{n}^{1}$, $R_{n}^{2}$, and $h_{n}$ of target *n* and *H* are assumed the same as Section 4.1. Firstly, there are 50% reckless targets with $d_{n}=5$ in the population of *N* and others are cautious with $d_{n}=1$. Secondly, the initial dynamic state $x_{n,0}$ and TEV state $P_{n,0}$ of each target *n* follow U(0,50) and U(0,40), respectively. The Whittle index truncates the corresponding infinite series to τ=16 terms with β=0.75. The simulation population is modified by letting *K* vary. The average performance of index policies and the lower bound across 1000 Monte Carlo simulations is shown in Figure 5, where the lower bound calculation is similar to the bound calculation with multi-dimensional TEC states in Section 3.3.

In Figure 5, the Whittle index policy also achieves the lowest discounted cost per target performance, which is stable at about 795. While the TEV index and myopic policies obtain 830 and 870, respectively. Consequently, the Whittle index policy obtains the closest performance to the lower bound. The simulation results of all the scenarios show the superiority of the Whittle index policies.

### 4.3. Performance Results with Multi-Dimensional States

This subsection attempts to validate the application of the US-MP index policy to the multi-dimensional states case. N=8 targets are tracked by the radar network with K=1,2,3 radars. The radars share the same parameters $P_{Tx}=1$ kw, $\varsigma =0.3^{\circ }$, and η=1 MHz. Without loss of generality, *K* radars are approximately in the same location $\left(x_{Tx},y_{Tx}\right)=\left(0,0\right)$. For each target, $\zeta_{n,t}=1$ during the radar tracking time horizon T=40 s. The MP index policy with the truncated time τ=6, the TEC index policy, and the myopic policy assess the tracking performance. In the US method when calculating the Whittle indices, γ=1.

The state transition matrices corresponding to the CV and CT model [41] are given by

(53) $$F_{n}^{1}=I_{2}⊗\left[\begin{array}{cc} 1 & T_{s}\\ 0 & 1 \end{array}\right],$$

(54) $$F_{n}^{2}=\left[\begin{array}{cccc} 1 & \frac{\sin\left(\omega T_{s}\right)}{\omega } & 0 & \frac{\cos\left(\omega T_{s}\right)-1}{\omega }\\ 0 & \cos\left(\omega T_{s}\right) & 0 & \sin\left(\omega T_{s}\right)\\ 0 & \frac{1-\cos\left(\omega T_{s}\right)}{\omega } & 1 & \frac{\sin\left(\omega T_{s}\right)}{\omega }\\ 0 & -\sin\left(\omega T_{s}\right) & 0 & \cos\left(\omega T_{s}\right) \end{array}\right],$$

where the tracking time interval $T_{s}$ is 1 s, and the turn rate is $3^{\circ }$/s. ⊗ represents the Kronecker product. $I_{2}$ denotes the 2×2 identity matrix.

The process noise covariance matrix is

(55) $$Q_{n}^{m}=q_{n}^{m}I_{2}⊗\left[\begin{array}{cc} T_{s}^{3}/3 & T_{s}^{2}/2\\ T_{s}^{2}/2 & T_{s} \end{array}\right],$$

where $q_{n}^{m}$ denotes the amplitude of the process noise.

For n=1,2,…,N, $q_{n}^{1}=10$, $q_{n}^{2}=20$, $R_{n,t}^{2}=R_{n,t}^{1}\circ \mathrm{diag}\left(4,2\right)$, and $R_{n,t}^{1}$ is calculated in (5) and (6). ∘ means the Hadamard product. The measurement cost $h_{n}=0$. Due to the fact that the smart target’s flight trajectory changes depending on the decision made at each time step, it is impossible to predefine the target trajectory. To address this, we use Monte Carlo simulation to thoroughly test a variety of flight trajectories and scenarios, yielding average performance results. The initial dynamic state estimation ${\hat{x}}_{n,0}$ and TEC state $P_{n,0}$ are generated by ${\left[50\cdot 10^{4},10^{2},50\cdot 10^{4},10^{2}\right]}^{\prime }\cdot \delta_{x}$ and $\mathrm{diag}\left(10^{4},10^{2},10^{4},10^{2}\right)\cdot \delta_{p}$, respectively, where $\delta_{x}∼U\left(0,1\right)$ and $\delta_{p}∼U\left(0.5,1.5\right)$. Three scenarios are defined for the performance analysis: one with reckless targets, one with cautious targets, and a third scenario that includes both types of targets, with four of each type. The weight is assumed as $d_{n}=5$, n=1,2,…,K, and $d_{n}=1$, n=K+1,K+2,…,N.

**Figure 6:**
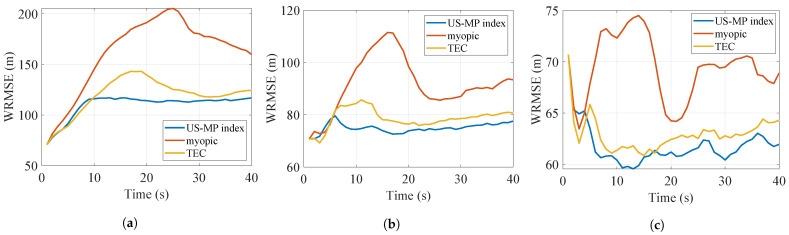
WRMSE of policies for reckless targets. (**a**) K=1 (**b**) K=2 (**c**) K=3.

**Figure 7:**
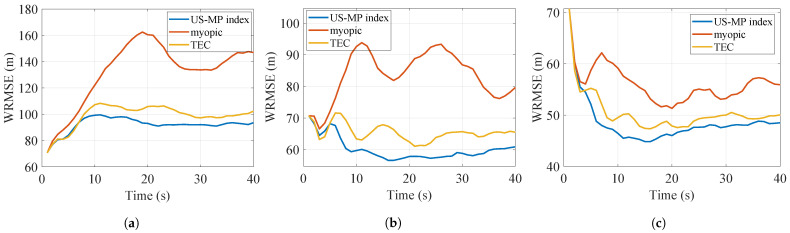
WRMSE of policies for cautious targets. (**a**) K=1 (**b**) K=2 (**c**) K=3.

**Figure 8:**
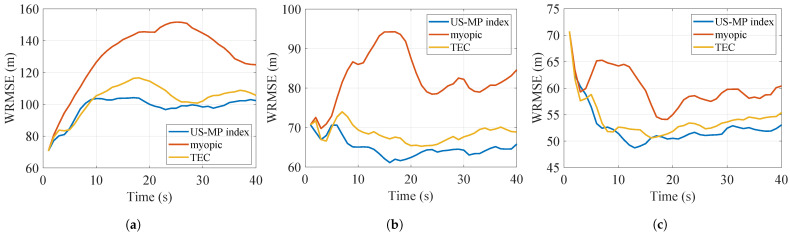
WRMSE of policies for reckless and cautious targets. (**a**) K=1 (**b**) K=2 (**c**) K=3.

The results with $N_{\mathrm{mc}}=1000$ Monte Carlo simulations are shown in Table 2. As the value of *K* varies from 1 to 3, the number of targets *N* remains constant at 8, and the number of targets tracked will increase. Consequently, the tracking costs and AWRMSEs obtained by all policies naturally decrease, and the difference between the performances of the US-MP index policy and other myopic policies decreases. The WRMSEs of the three scenarios are plotted in Figure 6, Figure 7 and Figure 8. The US-MP index policy based on the trace of TEC matrices always obtains lower tracking errors and outperforms the TEC index policy and myopic policy.

**Figure 9:**
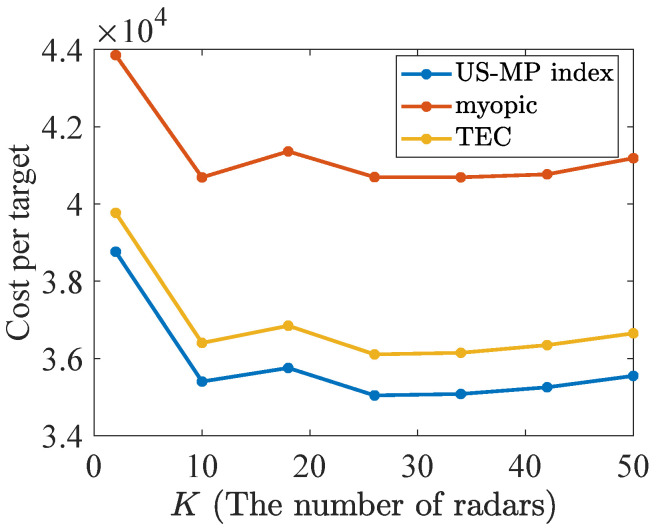
The discounted cost per target in the constant ratio 1/4.

**Figure 10:**
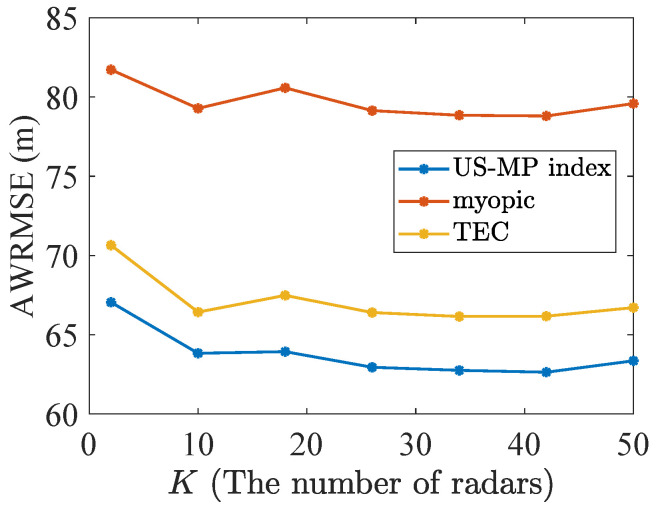
The AWRMSE in the constant ratio 1/4.

Here, the performances of policies are also analyzed while varying the number of targets and radars with a fixed ratio K/N=1/4. A scenario is considered to consist of 50% reckless targets with $d_{n}=5$ and 50% cautious targets with $d_{n}=1$, respectively. The discounted cost per target and AWRMSE results under $N_{\mathrm{mc}}=1000$ Monte Carlo simulations are shown in Figure 9 and Figure 10. As the number of radars and targets increases, the US-MP index policy obtains better tracking performance in all of the cases.

Above all, the adapted US-MP index policy improves the non-myopic beam scheduling performance and outperforms other greedy policies. Numerical simulation results with different parameters of the problem model indicate the superiority of this policy. However, the indexability validation and the lower bound need further calculations and verifications.

Additionally, for other optimization metrics, such as Bayesian CRLB [4], PCRLB [12], the associated state transition process can still be modeled using the US method. Therefore, the US-MP index policy can be extended to accommodate the new optimization objective functions.

## 5. Conclusions

This paper considered the beam scheduling problem of radar networks for multiple smart-target tracking, where the TEC and dynamic states of each target evolve based on the tracking action, the non-linear filter, and dynamic model probability parameters. The trade-off between more observation of smart targets for better tracking performance and the maneuvering reaction of smart targets to elude themselves was solved by the RMABP model based on the Whittle index policy. Through numerical simulation results for both scalar and multi-dimensional states, this paper has demonstrated the better performance of the US-MP index policy by comparing it with the myopic baseline policies. For future research, our plan is to prompt the joint resource scheduling for tracking smart targets through the MP index policy.

## Figures and Tables

**Table 1:** Performances with different numbers of radars for N=8 targets with scalar states.

Target Type	Policies	K=1		K=2		K=3	
		Cost	AWRMSE (m)	Cost	AWRMSE (m)	Cost	AWRMSE (m)
Reckless	Whittle index	**5138.56**	**26.57**	**4693.88**	**19.16**	**5027.96**	**19.59**
		(**16.44%**)	(**19.26%**)	(**17.23%**)	(**17.87%**)	(**14.54%**)	(**13.66%**)
	myopic	6149.71	32.91	5671.22	23.33	5883.68	22.69
		/	/	/	/	/	/
	TEV	5595.49	29.15	5115.57	20.79	5370.66	20.34
		(9.01%)	(11.43%)	(9.80%)	(10.89%)	(8.72%)	(10.36%)
Cautious	Whittle index	**3389.62**	**17.96**	**3112.04**	**12.95**	**3264.81**	**12.87**
		(**16.62%**)	(**22.75%**)	(**17.25%**)	(**19.96%**)	(**15.18%**)	(**16.37%**)
	myopic	4065.25	23.25	3760.86	16.18	3848.97	15.39
		/	/	/	/	/	/
	TEV	3597.30	19.65	3279.45	13.72	3403.97	13.29
		(11.51%)	(15.48%)	(12.80%)	(15.20%)	(11.56%)	(13.65%)
Reckless and Cautious	Whittle index	**4387.69**	**22.75**	**4096.84**	**17.58**	**4574.77**	**17.97**
		(**17.60%**)	(**21.23%**)	(**21.31%**)	(**21.87%**)	(**19.42%**)	(**21.08%**)
	myopic	5325.08	28.88	5206.35	22.50	5677.09	22.77
		/	/	/	/	/	/
	TEV	4841.00	25.19	4583.20	18.79	5048.45	19.39
		(9.09%)	(12.78%)	(11.97%)	(16.49%)	(11.07%)	(14.84%)

The bold numbers in the table highlight the optimal performance.

**Table 2:** Performances with different numbers of radars for N=8 targets with multi-dimensional states.

Target Type	Policies	K=1		K=2		K=3	
		Cost	AWRMSE (m)	Cost	AWRMSE (m)	Cost	AWRMSE (m)
Reckless	US-MP index	**269,150**	**110.09**	**219,078**	**74.89**	**195,336**	**61.78**
		(**16.84%**)	(**32.09%**)	(**12.48%**)	(**18.24%**)	(**9.32%**)	(**10.97%**)
	myopic	323,655	162.12	250,311	91.61	215,410	69.40
		/	/	/	/	/	/
	TEC	280,613	119.43	227,659	78.69	200,572	62.93
		(13.29%)	(26.33%)	(9.05%)	(14.10%)	(6.88%)	(9.32%)
Cautious	US-MP index	**274,512**	**92.07**	**217,766**	**60.05**	**193,309**	**48.61**
		(**13.32%**)	(**30.32%**)	(**13.08%**)	(**28.15%**)	(**7.47%**)	(**13.29%**)
	myopic	316,710	132.13	250,550	83.59	208,912	56.07
		/	/	/	/	/	/
	TEC	280,745	98.84	225,040	65.42	196,884	50.53
		(11.36%)	(25.19%)	(10.18%)	(21.73%)	(5.76%)	(9.88%)
Reckless and Cautious	US-MP index	**268,109**	**97.75**	**212,290**	**64.84**	**186857**	**52.69**
		(**14.89%**)	(**25.15%**)	(**12.60%**)	(**21.51%**)	(**8.76%**)	(**12.13%**)
	myopic	315,013	130.61	242,897	82.61	204,795	59.96
		/	/	/	/	/	/
	TEC	277,192	103.29	220,694	68.41	190,387	54.08
		(12.01%)	(20.91%)	(9.14%)	(17.19%)	(7.03%)	(9.81%)

The bold numbers in the table highlight the optimal performance.

## Data Availability

The raw data supporting the conclusions of this article will be made available by the authors on request.
